# Role of neutrophil to lymphocyte ratio as a prognostic indicator for COVID‐19

**DOI:** 10.1002/hsr2.442

**Published:** 2021-12-23

**Authors:** Samar A. Amer, Omar A. Albeladi, Arafa M. Elshabrawy, Naif H. Alsharief, Fatimah M Alnakhli, Amani F. Almugathaui, Samar S. Almashahadi, Hosam M Dawood, Muhammad Bilal Malik, Jaffer Shah, Hani Aiash

**Affiliations:** ^1^ Public Health and Community Medicine, Faculty of Human Medicine Zagazig University Zagazig Egypt; ^2^ Faculty of Medicine King Saud University, Ministry of Health (MOH) Riyadh Saudi Arabia; ^3^ Internal Medicine, Faculty of Human Medicine Zagazig University Zagazig Egypt; ^4^ Lab Department MOH Al‐ Madinah Al Monawarh Saudi Arabia; ^5^ Medical Science Faculty, Clinical Biochemistry Newcastle upon Tyne UK; ^6^ Department of Nursing Al‐Ghad International College for Medical Sciences, MOH Dammam Saudi Arabia; ^7^ Department of Nursing, Ministry of Health Tibah University Medina Saudi Arabia; ^8^ Tropical Department, Faculty of Human Medicine Zagazig University Zagazig Egypt; ^9^ Department of Internal Medicine SUNY Upstate Medical University Hospital Syracuse New York USA; ^10^ Kateb University Medical Center Kabul Afghanistan; ^11^ Department of Family Medicine Suez Canal University Ismailia Egypt; ^12^ Department of Cardiovascular Perfusion SUNY Upstate Medical University Syracuse New York USA; ^13^ Department of Interprofessional Research SUNY Upstate Medical University Syracuse New York USA

**Keywords:** COVID‐19 cases, NLR, outcomes, PCR, prognosis, virus load

## Abstract

**Background:**

Coronavirus disease 2019 (COVID‐19) is a new pandemic disease, associated with substantial morbidity and mortality. Its diagnosis requires centralized facilities and time.

**Aims:**

To describe the exposure history and clinical picture of the COVID‐19 patients, to study the SARS‐CoV‐2 Virus load and some determinants that may correlate with its prognosis, and to evaluate the role of inflammatory index NLR as an early predictor of COVID‐19 prognosis.

**Methodology:**

A prospective follow‐up study included laboratory‐confirmed 179 COVID‐19 cases out of 660 suspected COVID‐19 cases, at El‐Madinah El‐Monawarah General Hospital in April 2020. Confirmed cases were managed by the Saudi Protocol and followed up every 2 weeks by PCR, neutrophil to lymphocyte ratio (NLR) for 1 month. Data were collected through a validated questionnaire and by qualified infection control staff.

**Results:**

The majority of the COVID‐19 cases were 67 (37.4%) aged 30 to <45 years, 157 (87.7%) males, 76.0% working outside the medical field. 38.0% were asymptomatic and 26.3% had severe symptoms, while the main presenting symptoms were fever and dry cough (49.7% and 43.6%), respectively. The case fatality was 7.8%. The male, nonmedical occupation, and low level of education had a statistically significant relationship with the baseline PCR. There was an inverse significant correlation between baseline PCR readings and the recovery duration and health status outcomes. NLR was noted to be significantly higher among old age, illiterate nonmedical occupation, case with severe symptoms, MICU admission, and worst health status outcomes, but it was paradoxically higher among nonadmitted positive cases.

**Conclusion:**

Admitted COVID‐19 cases outcomes (disease severity, ICU admission, and mortality) significantly correlated to NLR and not to the baseline PCR viral load. NLR could be a beneficial prognostic and triaging parameter especially old nonmedical COVID‐19 patients.

**Abbreviations**
ARDS: acute respiratory distress syndrome
CAP: community‐acquired pneumonia
COVID‐19: Coronavirus disease 2019
FETP: field epidemiology training program
HAP: hospital‐acquired pneumonia
MICU: Medical intensive care unit
MOH: Ministry of Health
NLR: neutrophil/lymphocyte ratio
PCR: polymerase chain reaction
RD: respiratory distress
RF: respiratory failure
RR: Respiratort Rate
SARI: severe acute respiratory illness
SARS‐CoV‐2: severe acute respiratory syndrome coronavirus 2
SMOP: Saudi Ministry of Health Protocol

## 1 INTRODUCTION

Coronavirus disease 2019 (COVID‐19) is a new pandemic disease,^1^ associated with substantial morbidity and mortality.^2^ Its clinical spectrum is very heterogeneous, and the leading cause of death is the respiratory failure from acute respiratory distress syndrome (ARDS).^3^ COVID‐19 transmission occurs through respiratory droplets, direct contact,^4^, ^5^ nosocomial,^1^, ^3^ and airborne in healthcare settings during aerosol‐generating procedures and some community outbreaks.^6^ Super spreading events can pass the infection to large numbers of contacts^7^ and are associated with explosive growth early in an outbreak and sustained transmission in later stages.^8^

The SARS‐CoV‐2 RNA virus shedding was observed in mild and severe cases.^9^ The shedding duration is longer in stool samples than in respiratory and serum samples {22 days (d), vs 18 days, 16 d}^10^ and in symptomatic than asymptomatic patients (25.2 days vs 22.6 days).^11^ Factors associated with prolonged shedding include older age, male sex, comorbid hypertension, severe illness on admission or delayed hospital admission after symptom onset, use of corticosteroids, and invasive mechanical ventilation.^12^, ^13^ The virus load peaks within the first week of disease onset^14^ and detected in high levels in nasal and throat swabs after symptom onset and be nearly similar in asymptomatic and symptomatic patients.^15^, ^16^ Baseline high viral load may increase the risk of disease progression.^17^

The virus particles invade the lymphocyte cytoplasmic component and cause destructive necrosis or apoptosis.^18^ Therefore, the severity of lymphocytopenia reflects the severity of infection. Neutrophil to lymphocyte ratio (NLR) is a meaningful parameter for prognosis and risk state and can help to appropriately allocate medical resources.^19^ The prognostic factors that increase the risk of unfavorable outcomes include male sex, age ≥ 50 year, comorbidities (eg, hypertension, diabetes), smoking, lymphopenia, thrombocytopenia, liver or renal impairment, cardiac injury, elevated inflammatory markers (C‐reactive protein, ferritin), elevated D‐dimer, and elevated interleukin‐6.^20^ Most of these findings are a result of studies initially performed in China, and additional validation studies, especially outside of China, are required.^21^

There is increasing evidence that easily obtained laboratory biomarkers, such as NLR, can have a predictive role and can be used to stratify patient risk and individualize treatment strategy in a variety of medical conditions. Such laboratory tests are being used in conditions such as acute coronary syndrome,^22^ cerebral hemorrhage,^23^ ischemic stroke,^24^, ^25^ and sepsis, and other infectious pathologies.^26^

We aim to study the SARS‐CoV‐2 Virus load and explore some determinants that affect its prognosis, through the following objectives and a hospital‐based study in Saudi Arabia;

1. To describe the exposure history and clinical picture of the COVID‐19 patients.
2. To study the relationship between SARS‐CoV‐2 virus load and the different demographic determinants that may correlate with its prognosis.
3. To evaluate the role of inflammatory index NLR as an early predictor of COVID‐19 prognosis.

## 2 METHODOLOGY

### 2.1 Study design and participants

**FIGURE 1:**
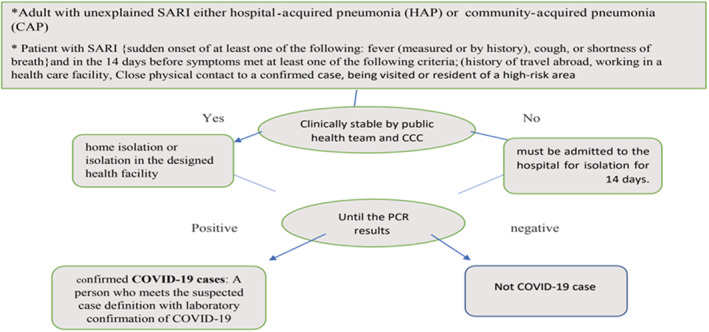
The Saudi Ministry of health protocol (SMOP) with the suspected coronavirus disease 2019 (COVID‐19) cases (22), tested for COVID‐19 polymerase chain reaction (PCR)

This prospective cohort study targeted all referred cases to El‐Madinah El‐Monawarah General Hospital (main governmental hospital in the western region, kingdom of Saudi Arabia) of 686 participants, during April 2020. The referred cases according to the Saudi Ministry of health Protocol (SMOP) as a suspected COVID‐19 cases.^27^ Tested for COVID‐19 PCR as in Figure 1.

### 2.2 Sample size

We recruited all the admitted cases.

### 2.3 Data collection through the

Data collection team: COVID‐19 management team through

- Face‐to‐face interview by the infection control team to collect the demographic characteristics, the exposure history, and the clinical assessment of the presenting Symptoms and occurance of complications.
- Sample collection through a well‐strained GP and the laboratory team
- Lab sheet (record base) for results of the collected samples.

#### 2.3.1 The data collection tool

The questionnaire was designed on Arabic Google forms was used to collect data, after validated by nine experts for validating its content and its reliability and then Pilot testing was done and involved 25 participants in order to ensure clarity of the questionnaire, and results of the pilot were not included in the study. The questionnaire consisted of main parts:

1. The demographic characteristics of the studied cases.
2. The exposure history including source, date of infection and the spreading events.
3. The clinical profile;
  - Symptoms and complications.
  - Laboratory finding including
    Baseline neutrophil, lymphocyte, and NLR,
    PCR three reading (repeated every 2 week till become negative for 1 month)
4. Follow up; to assess the prognosis or current health status (at time of the data collection) including duration until recovery, or/ discharge.

#### 2.3.2 The study variables

- *Respiratory failure (RF)* is a syndrome in which the respiratory system fails in one or both of its gas exchange functions: carbon dioxide elimination and oxygenation, classified as either hypercapnia or hypoxemic^28^
- *Respiratory distress (RD)* is a sudden onset respiratory failure in adults that follows lung endothelium injury lung (as in sepsis or pneumonia) with the accumulation of protein‐rich fluid and alveoli collapse leading to difficult, rapid breathing, and very low levels of oxygen in the blood^28^
- *Discharging protocol* to discontinue isolation precaution, two respiratory negative samples 24 hours apart are required for all patients
- *Patients were retested by polymerase chain reaction (PCR); (according to the updated SMOHP*,^27^ in symptomatic cases who are isolated in hospitals, when the patient is clinically free, or if the result is positive, repeat the test every 72 hours.
- *PCR for diagnosing COVID‐19 virus*; according to WHO guidelines,^29^ was measured through collecting nasopharyngeal swabs by qualified well‐trained medicinal specialists from both the left and right nasal cavities of the same patient. Then, the sample was kept in a sample collection tube containing 3 mL of standard viral transport medium. Based on the results, participants were classified into;
  - Negative PCR samples; means free from COVID‐19 cases, who were excluded,
  - Positive PCR samples; means confirmed cases, who will recruited in the study.
- *NLR* was collected by complete blood count (CBC) by nurses through venous collection; blood was collected in K2 EDTA 3 mL tube (IMPROVE by Guangzhou, Improve Medical Instruments, Guangdong, China) according to standard venous blood collection protocol using vacutainer system.
- *The viral load* is the amount or concentration of a virus in a given quantity in body fluid, often expressed as the number of viral particles per milliliter of the fluid: estimated with the DCt method (CT sample–Ctref) for diagnosed positive cases and were analyzed in the main lab in Madinah.

#### 2.3.3 Statistical analysis

The collected data were coded and analyzed by using SPSS (version 22) at the level of significance (*P*‐value ≤ .05). For quantitative data, summarization means, SD, median, and range were used, while Kruskall–Wallis *U*‐test and analysis of variance (ANOVA) f‐test were used for their analysis. To test the association between categorical variables, Pearson's correlation coefficient (r) was used to test the association between two continuous variables.

### 2.4 Ethical issues

The questionnaire contains no sensitive or private questions. Ethical approval form the committee of the research center at King Fahad Medical City. IRB Registration Number with KACST, KSA: H‐01‐R‐012.

### 2.5 Patient and public involvement

The data were collected after informed consent from the participants' records, and direct examination, and their identity was anonymous.

## 3 RESULTS

A total of 686 patients who were referred to Al Madinah El Monawarah hospital to be examined were tested with COVID‐19 PCR, 73.9% were negative, 179 (26.1%) were positive {82 (45.8%) symptomatic with hospital isolation, 97 (54.2%) asymptomatic isolated at hotel}.68 (38%) were asymptomatic, and 111 (62.0%) of case were symptomatic

- The case fatality rate in this setting on April =14/179*100 = 7.8%
- The percentage of confirmed cases in this setting on April =179/686 = 26%
- The percentage of severe and critical cases = 47/179 = 26.3%

Among the studied COVID‐19 cases, the majority 123 (68.7) did not know the date of exposure and only 87 (48.6) reporting a history to a confirmed case. Work was the main reported spreading event for COVID‐19 infection (Table 1).

**TABLE 1:** The exposure history to COVID‐19 infection among patients

	F (%)
History of contact (T = 179)	
Yes, to a confirmed case	87 (48.6)
No/do not know	92 (51.4)
Spreading events (site) (T = 179)	
Do not know	41 (22.3)
Work	59 (32.4)
House	32 (17.9)
Abroad^a^	4 (1.7)
Work + house	24 (12.8)
Health care centers	15 (8.3)
Malls/markets	4 (1.7)
The data of exposure (T = 179)	
Do not know	123 (68.7)
Within less than 5 days	50 (27.9)
5 days‐less than 10 days	4 (1.3)
10 days or more	2 (1.1)

^a^ The KSA closed its airports on (1 March).

Totally, 136 (75.9%) of COVID‐19 patient had no co‐morbidities, 68 (38.0) were asymptomatic, the main presenting symptoms were fever 89 (49.7), dry cough 78 (43.6), difficulty in breath 55 (30.7) in descending order, and 47 (26.3%) presented with severe symptoms (Table 2).

**TABLE 2:** The clinical symptoms and course of the COVID‐19 cases

	F (%)
Symptoms^a^	
No symptoms	68 (38.0)
Nausea–vomiting	8 (4.5)
Fever	89 (49.7)
Dry cough	78 (43.6)
Difficulty breath	55 (30.7)
Sore throat	35 (19.6)
Headache	5 (2.8)
Fatigue	6 (3.4)
Muscle pain	1 (0.6)
Corneal affection	0 (0,0)
Diarrhea	5 (2.8)
Nasal congestion	3 (1.8)
Others	4 (2.2)
Sever symptoms	47 (26.3)
O_2_ saturation < 93%	44 (24.6)
Respiratory distress > 30b/min	3 (1.7)
Respiratory failure	3 (1.7)
The interval between the appearance of symptoms and the diagnosis by PCR (T = 111)	
Less than 3 days	50 (45.0)
3 days to <1 week	39 (35.1)
1 week or more	22 (19.8)
The needed management procedures^b^	
Inpatient	49 (27.4)
MICU	8 (4.5)
Hotel isolation	128 (71.5)
Self‐isolation	16 (8.9)
The duration of the received care (inpatient and ICU)	
<1 week	14 (7.8)
1 week to <2 week	21 (11.7)
2 week to <3 week	11 (6.1)
3 week or more	11 (6.1)
The current health status of cases (T = 179)	
Deaths	13 (7.3)
Stable cases under medication	15 (8.4)
Stable cases without medications	83 (46.4)
Turned negative but still hospitalized	2 (1.1)
Discharged after Turned negative	66 (36.9)
Duration from diagnosed till negative PCR (d)	21.0 ± 8.6 (5‐39)

Abbreviation: MICU, medical intensive care unit.

^a^ Multiple answers were allowed.

^b^ As cases followed for month, multiple care procedures were allowed depending on the cases prognosis.

The majority of the COVID‐19 cases were aged 30 to < 45 years (67 (37.4%)), 157 (87.7%) males, 88.8% non‐Saudi, 126 (70.4%) single, 100 (55.9%) secondary or high school, and 136 (76.0%) working outside the medical field. The virus load is significantly higher in single, male, illiterate, and people working outside the medical field (Table 3). 87 (48.6%) had a history of contact with a confirmed case, in which 58 (32.4%) work was the main site of spreading, and 123 (68.7%) did not know the date of exposure (Table 1).

**TABLE 3:** The demographic characteristics of COVID‐19 cases and its relationship with the baseline main laboratory investigations

		first PCR Mean ± SD	Lymphocyte median (mean ± SD)	Neutrophil median (mean ± SD)	NLR median (mean ± SD)	Recovery duration (d) M (mean ± SD)
	F (%)/total	29.1 ± 4.8 (16.8‐38.3)	1.5 (2.34 ± 7.9) (0.26‐73)	3.8 (5.34 ± 3.5) (0.87‐17.28)	2.98 (4.89 ± 4.87) (0.09‐24.47)	21.0 ± 8.6 (5‐39)
Age groups:						
15 to <30 years	3	29.3 ± 4.4	1.8 ± 0.6	3.2 (3.2 ± 2.2)	1.4 (1.9 ± 1.5)	21.5 (19.6 ± 10.8)
30 to <45 years	67 (37.4)	29.1 ± 4.9	1.4 ± 0.6	3.6 (5.5 ± 3.9)	2.9 (5.1 ± 5.2)	17 (19.5 ± 9.7)
45 to <60 years	44 (24.6)	28.9 ± 5.1	3.4 ± 1.5	4.1 (5.6 ± 3.4)	3.2 (4.6 ± 4.4)	22 (22.6 ± 7.2)
60 to <75 years	16 (8.9)	28.0 ± 4.4	1.19 ± 0.6	4.6 (5.9 ± 3.3)	3.7 (6.9 ± 6.0)	21 (21.6 ± 7.0)
75 years or more	3 (1.7)	29.5 ± 5.3	0.7 ± 0.11	6.6 ± 2.6	9.2 (9.2 ± 5.2)	23
*P*		.59	.62	.37	.04*	.55
Sex						
Female	22 (12.3)	27.2 ± 5.3	1.46 (1.6 ± 0.75)	3.6 (4.7 ± 3.3)	2.5 (3.9 ± 2.9)	22.5 ± 9.4
Male	157 (87.7)	29.3 ± 4.6	1.41 (1.52 ± 0.9)	4.09 (5.5 ± 3)	3.3 (5.3 ± 5.2)	20.6 ± 8.4
*P*		.04*	.66	.39	.18	.44
Nationality						
Saudi	20 (11.2)	25.1 ± 6.0	1.9 (1.9 ± 0.7)	3.7 (4.5 ± 3.3)	2.29 (3.0 ± 3.1)	28 (25 ± 10.8)
Egyptian	13 (7.3)	27.6 ± 6.6	1.5 (1.4 ± 0.5)	3.1 (4.2 ± 2.9)	2.4 (2.8 ± 1.2)	16.5 (16.3 g ± 4.5)
Others	146 (81.6)	29.7 ± 4.1	1.4 (1.4 ± 1.0)	4.3 (5.8 ± 3.5)	3.5 (5.7 ± 5.4)	21 (20.7 ± 8.1)
*P*		.00*	.89	.26	.05*	.06
Level of education						
Illiterate	8 (4.5)	27.6 ± 5.9	1.3 (1.29 ± 0.7)	5.7 (5.8 ± 1.8)	4.7 (6.6 ± 5.1)	20 ± 7.9
Read and write/primary	51 (28.5)	29.4 ± 4.4	1.39 (1.49 + −1.4)	4.06 (4.9 ± 2.7)	3.7 (5.2 ± 5.1)	20.8 ± 8.4
Secondary/high school	100 (55.9)	29.7 ± 4.2	1.65 ± 0.8	3.7 (6 ± 4.3)	3 (5.1 ± 5.5)	20.0 ± 7.7
University or above	20 (11.2)	25.2 ± 5.8	1.44 ± 0.5	3.8 (4.2 ± 2.4)	2.3 (3.5 ± 2.9)	22.8 ± 10.3
*P*		.00*	.55	.32	.53	.77
Marital status						
Married	53 (29.6)	28.0 ± 5.3	1.4 (1.5 ± 1.1)	3.9 (5.03 ± 2.9)	2.9 (4.7 ± 4.3)	21 (21.8 ± 8.1)
Single	126 (70.4)	29.4 ± 4.4	1.5 (1.6 ± 0.7)	3.5 (5.7 ± 3.9)	3.3 (5.2 ± 5.4)	20.5 (20.1 ± 9.1)
*P*		.06	.39	.40	.69	.41
Occupation						
Not working	31 (17.3)	27.5 ± 5.1	1.2 (1.47 ± 0.9)	3.5 (4.6 ± 3.4)	2.9 (4.9 ± 4.9)	24.3 ± 7.8
Working in the medical field	12 (6.7)	24.8 ± 6.3	1.5 (1.58 ± 0.3)	3.7 (4.5 ± 2.4)	2.5 (3.1 ± 1.9)	22.6 ± 10.11
Working outside the medical field	136 (76.0)	29.8 ± 4.3	1.41 (1.6 ± 1.0)	4.6 (5.8 ± 3.6)	3.2 (4.9 ± 4.3)	19.1 ± 8.1
*P*		.00*	.87	.37	.03*	.19

Abbreviations: NLR, neutrophil/lymphocyte ratio; PCR, polymerase chain reaction.

^*^ *P* < .05 there is a statistically significant difference.

There were a statistically significant (*P* < .05) relationship between the type of comorbidities and the median of lymphocyte, median of NLR, and the recovery duration (d). The highest recovery duration was significantly higher among COVID‐19 cases with companied comorbidities (31.5 ± 7.4). The median of NLR was significantly higher among infected patients who had DM and HTN 5.7 (6.6 ± 4.7), them among diabetic patients 3.8 (5.6 ± 5.7) compared to other groups (Table 4).

**TABLE 4:** The relationship between the comorbidities and some laboratory findings, recovery duration, and the occurrence of death

	NLR median (range)	Lymphocyte median (range)	Neutrophil median (range)	PCR Mean ± SD	Recovery duration (d) mean ± SD	Death No = 13 No (%)
No diseases	2.8 (4.84 ± 5.1)b	1.5 (2.8 ± 9.9)b	3.9 (5.6 ± 3.9)	29.3 ± 4.8	(20.9 + 8.9)b	7 (53.8)
Diabetes Mellitus (DM)	3.8 (5.6 ± 5.7)a	1.5 (1.45 ± 1)b	3.4 (4.9 ± 3.5)	28.3 ± 4.7	(21.3 ± 5.5)b	1 (7.6)
Hypertension HTN	2.5 (3.1 ± 1.4)b	1.7 (1.8 ± 0.6)	3.9 (6.1 ± 5.0)	28.5 ± 2.4	(19.2 ± 8.4)b	0 (0.00)
DM + HTN	5.7 (6.6 ± 4.7)c	0.82 (0.99 ± 0.6)a	4.5 (5.2 ± 2.8)	27.1 ± 5.	(21.4 ± 6.9)b	4 (30.0)
Others/companied	1.5 (2.3 ± 1.9)d	1.7 (1.8 ± 0.6)	3.2 (3.8 ± 1.5)	29.1 ± 6.3	(31.5 ± 7.4)a	1 (7.6)
*P*	.00*	.01*	.80	.64	.00*	

*Note*: The alphabet of different symbols ‐ shows statistical significant difference.

Abbreviations: NLR, neutrophil/lymphocyte ratio; PCR, polymerase chain reaction.

^*^ *P* < .05 there is a statistically significant difference.

The sex (*P* = .04*), occupation (*P* = .00*), and level of education (*P* = .00*) had statistically significant relationship with the baseline PCR, whereas the NLR is significantly related to the age groups (*P* = .04*), occupations (*P* = .03*), and nationality (*P* = .05*).

## 4 DISCUSSION

It is very important to grade the severity of COVID‐19 infection for treatment, especially in outbreak or epidemic, because the medical resources are relatively scarce, optimize the allocation of rescue resources, and prevent the occurrence of overtreatment or under treatment.^30^ In this study, we report here a prospective study on 179 patients with laboratory‐confirmed COVID‐19 infection.

Work was the main source of catching the history of exposure, and Doaa et al.^31^ reported nearly similar main clinical symptoms because both samples had nearly similar demographic characteristics.

The viral load was found to be significantly higher (*P* = .00*) in patients who worked outside the medical field than those working in the medical field or not working; this can be explained by the protective measures and the higher awareness of the medical personnel.

In addition, it was significantly lower in highly educated than others; this may spotlight the importance of awareness campaign. It was higher in non‐Saudi patients; this may be due to different immune responses and vaccination programs. In addition, it was higher in males than females; this can be explained by higher exposure rates in males than females in Saudi Arabia.

Fifteen percentage of patients were diabetic as it is a risk factor for severe disease,^32^ hospitalization, and mortality,^33^ due to the impaired in the immune response.^34^ Nearly, similar findings (22%, 16.2%, and 12%) were reported in many studies (30.31, 29) in order. 11% (19) had hypertension. Forty‐nine percentage had a history of exposure or contact with a confirmed case, 32% of our patients most likely contracted the infection at the workplace while 18% at home. Only 3% were infected abroad, but it has to be noted that Saudi Arabia closed its airports on March 1, before the inclusion period of this study.

Fever was present in 89 (50%) and dry cough in 78 (44%); these were the main presenting symptoms in most of our patients; this in agreement with Yousef et al, in their multicenter study in Saudi Arabia.^35^ These features bear some resemblance to SARS‐CoV and MERS‐CoV infections.^32^, ^36^

There were a statistical significant (*P* < .05) relationship between the type of comorbidities and the median of lymphocyte, median of NLR, and the recovery duration (d). The NLR was significantly higher among COVID‐19 infected patients who had DM and HTN 5.7 (6.6 ± 4.7), them among diabetic patients 3.8 (5.6 ± 5.7) compared to other groups (Table 4). DM has a significant relation with the morbidity and mortality of the affected person that increase the burden on health care system.^37^

Severe symptoms including O2 saturation < 93%, Respiratory distress (RR >30b/min), or Respiratory failure occurred in about 50% of our patients. The interval between the appearance of symptoms and the diagnosis by PCR was less than 1 week in about 80%, and less than 3 days in 45%, which is consistent with other studies.^38^, ^39^, ^40^ Hospitalization was needed in about one‐third of our patients, with ICU admission in only eight patients (4.5%) while hotel isolation in 128 patients (71.5%) (Table 5).

**TABLE 5:** The virus load changes among the studied cases

	PCR 1 st reading	PCR second reading F (%)	PCR third reading F (%)
Mean ± SD	(29.1 ± 4.8)	0 (10.9 ± 15.3)	0 (4.2 ± 11.2)
Range	(16.67‐38.29)	0‐38	(0‐39)
No of positive cases	No =179	62 (34.6%)	25 (13.9)
Turned negative		117 (65.4%)	154 (86.1)
Mean ± SD	(29.1 ± 4.8)	31.8 ± 3.5	32.5 ± 2.5
Range	(16.67‐38.29)	18.4‐38.5	25.2‐39
No of Positive cases	No = 179	N0 = 62	No = 25

In the present study, a negative correlation was found between viral load (first PCR) with duration until recovery and severity of the disease (Table 6). This may be contradictory with what is known of most viral infection for example, influenza,^41^ this can be explained by the difference in virus strain or early discovery due to early presentation. This is contradictory with Paolo Cotzia who said: “It appears that the viral load peaks in the early stages of the disease. Although it is not associated with the duration of symptoms, their severity or outcome, it appears that the viral load is an important epidemiological surrogate marker of infectivity in mildly symptomatic and asymptomatic non‐hospitalized patients.”^42^ This is also contradictory with Xia et al, who found a positive correlation between sputum viral load with disease severity and risk of progression^17^ but this in agreement with recent updates published by Medscape.com ^43^ that patients with higher viral loads of COVID‐19 were less likely to require hospital admission. Investigators studied 205 adults with confirmed COVID‐19 in the emergency department at New York University Langone Medical Center, took nasopharyngeal swabs, and measured SARS‐CoV‐2 viral load using RT‐PCR assays (Table 7).^43^

**TABLE 6:** The relationship between the patient outcomes and the baseline main laboratory investigations

	NLR median (range)	Lymphocyte median (range)	Neutrophil median (range)	PCR mean ± SD	Recovery Duration (d) Median (range)
Inpatient					
No	14 (14.1 ± 14.7)	0.83 (0.83 ± 0.3)	9.3 (9.25 ± 7.7)	27.4 ± 6.5	18 (21.3 ± 7.5)
Yes	3.8 (5.8 ± 4.8)	1.2 (1.39 ± 0.7)	4.7 (6.23 ± 3.9)	28.1 ± 4.9	22 (22.1 ± 7.9)
*P*	.00*	.45	.01*	.79	.45
MICU					
No	2.8 (4.25 ± 4.1)	1.5 (1.6 ± 0.9)	3.7 (4.8 ± 3.0)	29.4 ± 4.7	21 (20.5 ± 8.1)
Yes	8.4 (9.6 ± 5.1)	1.2 (1.4 ± 0.8)	9.1 (10.3 ± 2.5)	25.3 ± 4.3	30.4 ± 2.1 +6 deaths
*P*	.00*	.85	.00*	.05	.00*
The current health status of cases^a^					
Stable cases under medication	2.8 (3.1 ± 2.0)a	1.7 (1.6 ± 0.7)	3.4 (4.1 ± 2.1)a	(29.2 ± 4.7)a	
Turned negative	2.5 (3.9 ± 4.0)a	1.5 (3.3 ± 11.0)	3.8 (4.9 ± 3.1)a	(28.1 ± 4.9)a	21 ± 8.3
Stable cases without medications	2.9 (5.2 ± 4.8)b	1.16 (1.4 ± 0.7)	3.9 (5.3 ± 3.8)a	(30.1 ± 4.4)a	
Deaths	8.4 (10.2 ± 6.9)c	0.68 (−.99 ± 0.59)	8.8 (8.3 ± 3.9)b	(25.9 ± 4.3)b	
*P*	.001*	.76	.024*	.01*	
The severe symptoms					
No	2.3 (3.3 ± 3.2)c	1.6 (1.6 ± 0.7)	3.5 (4.2 ± 2.7)c	29.1 ± 4.9	21.1 ± 8.2
O_2_ saturation < 93%	4.3 (6.5 ± 5.3)b	1.2 (3.6 ± 12.9)	5.4 (6.3 ± 3.7)c	29.0 ± 4.5	20.9 ± 7.2
O_2_ saturation < 93% + RF	6.4 (6.4 ± 2.9)b	1.6 (1.6 ± 0.8)	9 (9.0 ± 0/34)b	28.4 ± 2.2	DIED
O_2_ saturation < 93% + RD	16.3 (16.3_11.4)a	0.99 (0.99 ± 0.55)	13 (13.0 ± 2.3)a	23.8 ± 0.3	DIED
*P*	.00*	.76	.00*	.29	.92

*Note*: The alphabet of different symbols ‐ shows statistical significant difference.

Abbreviations: MICU, medical intensive care unit; NLR, Neutrophil/lymphocyte ratio; PCR, polymerase chain reaction; RD, respiratory distress; RF, respiratory failure.

^a^ The current health status (at 30th April) during the data of data collection during the follow‐up.

^*^ *P* < 0.05 there is a statistically significant difference.

**TABLE 7:** The correlation between the first baseline PCR and the following variables among COVID‐19 cases

The first baseline PCR reading and	r	*P*
Lymphocyte	0.15	.10
Neutrophil	0.01	.94
Second PCR reading	−0.12	.19
Recovery duration (d)	−.31	.01*
NLR	−0.06	.61
Age (y)	−0.09	.24

*Note*: r for spearman correlation coefficient test.

Abbreviations: NLR, neutrophil/lymphocyte ratio; PCR, polymerase chain reaction.

^*^ *P* < .05, there is a statistically significant difference.

We found no correlation between the baseline PCR with Lymphocyte percent, Neutrophil, second PCR, NLR, or age. A strong positive correlation between NLR and neutrophil count with disease severity, ICU admission, and mortality was found, this is in agreement with Jingyuan Liu who found that NLR is a good predictor for COVID‐19 critical illness in the early stage of the disease.^44^ Also, in agreement with Yang et al and Xisheng who stated that high NLR cab is considered as an independent bad prognostic biomarker.^45^, ^46^ Although all this we found that NLR was lower in patients admitted to the hospital when compared to patients who received home treatment.

## 5 CONCLUSION

The admitted COVID‐19 cases outcomes (disease severity, ICU admission, and mortality) correlated well to the NLR and not to the baseline PCR viral load. NLR could be a beneficial prognostic and triaging parameter for the admitted cases especially old non‐medical patients.

### 5.1 Limitations

It is difficult to assess other host risk factors for disease severity and mortality with multivariable‐adjusted methods.

### 5.2 Recommendation

Further detailed research studies are still required to verify and refine our findings to help develop a practical and effective tool for predicting disease outcomes in COVID‐19 patients.

## FUNDING

None declared.

## CONFLICT OF INTEREST

The authors reported no potential conflict of interest.

## TRANSPARENCY STATEMENT

I affirm that this manuscript is an honest, accurate, and transparent account of the study being reported; that no important aspects of the study have been omitted; and that any discrepancies from the study as planned (and, if relevant, registered) have been explained.

## AUTHOR'S CONTRIBUTIONS

Conceptualization: Samar Amer, Omar A. Albeladi, Naif H. Alsharief.

Data Curation: Samar A. Amer, Omar A. Albeladi, Arafa M. Elshabrawy, Amani F. Almugathaui, Samar S. Almashahadi, Naif H. Alsharief, Jaffer Shah.

Formal Analysis: Samar A. Amer, Arafa M. Elshabrawy, Naif H. Alsharief, Hosam M Dawood, Jaffer Shah.

Funding Acquisition: Omar A. Albeladi, Fatimah M Alnakhli, Amani F. Almugathaui.

Investigation: Samar A. Amer, Arafa M. Elshabrawy, Naif H. Alsharief, Fatimah M Alnakhli, Amani F. Almugathaui, Muhammad Bilal Malik, Hani Aiash.

Methodology: Samar A. Amer, Arafa M. Elshabrawy, Naif H. Alsharief, Fatimah M Alnakhli, Amani F. Almugathaui, Hosam M Dawood, Jaffer Shah, Hani Aiash.

Project Administration: Samar A. Amer, Omar A. Albeladi, Fatimah M Alnakhli, Hani Aiash.

Software: Jaffer Shah.

Supervision: Samar A. Amer,Omar A. Albeladi, Hani Aiash.

Validation: Samar A. Amer, Omar A. Albeladi.

Visualization: Hosam M Dawood, Naif H. Alsharief, Jaffer Shah.

Writing – Original Draft Preparation: Samar A. Amer, Arafa M. Elshabrawy, Samar S. Almashahadi, Hosam M Dawood, Jaffer Shah.

Writing – Review & Editing: Samar A. Amer, Samar S. Almashahadi, Hosam M Dawood, Jaffer Shah, Hani Aiash.

All authors have read and approved the final version of the manuscript. Samar A. Amer had full access to all of the data in this study and takes complete responsibility for the integrity of the data and the accuracy of the data analysis.

## Data Availability

The datasets used and/or analyzed during the current study are available from the corresponding author on reasonable request. No additional data available.
